# *N*-Carbamoylputrescine Amidohydrolase of *Bacteroides thetaiotaomicron*, a Dominant Species of the Human Gut Microbiota

**DOI:** 10.3390/biomedicines11041123

**Published:** 2023-04-07

**Authors:** Hiromi Shimokawa, Mikiyasu Sakanaka, Yuki Fujisawa, Hirokazu Ohta, Yuta Sugiyama, Shin Kurihara

**Affiliations:** 1 Faculty of Bioresources and Environmental Sciences, Ishikawa Prefectural University, Nonoichi 921-8836, Ishikawa, Japan; 2 Faculty of Biology-Oriented Science and Technology, Kindai University, Kinokawa 649-6493, Wakayama, Japan

**Keywords:** human gut microbiota, polyamine, *N*-carbamoylputrescine amidohydrolase, putrescine, agmatine, spermidine, *Bacteroides thetaiotaomicron*

## Abstract

Polyamines are bioactive amines that play a variety of roles, such as promoting cell proliferation and protein synthesis, and the intestinal lumen contains up to several mM polyamines derived from the gut microbiota. In the present study, we conducted genetic and biochemical analyses of the polyamine biosynthetic enzyme *N*-carbamoylputrescine amidohydrolase (NCPAH) that converts *N*-carbamoylputrescine to putrescine, a precursor of spermidine in *Bacteroides thetaiotaomicron,* which is one of the most dominant species in the human gut microbiota. First, *ncpah* gene deletion and complemented strains were generated, and the intracellular polyamines of these strains cultured in a polyamine-free minimal medium were analyzed using high-performance liquid chromatography. The results showed that spermidine detected in the parental and complemented strains was depleted in the gene deletion strain. Next, purified NCPAH-(His)_6_ was analyzed for enzymatic activity and found to be capable of converting *N*-carbamoylputrescine to putrescine, with a Michaelis constant (*K*_m_) and turnover number (*k*_cat_) of 730 µM and 0.8 s^−1^, respectively. Furthermore, the NCPAH activity was strongly (>80%) inhibited by agmatine and spermidine, and moderately (≈50%) inhibited by putrescine. This feedback inhibition regulates the reaction catalyzed by NCPAH and may play a role in intracellular polyamine homeostasis in *B. thetaiotaomicron*.

## 1. Introduction

Polyamines are aliphatic amines with two or more amino groups and are found in almost all living organisms, from prokaryotes to higher plants and animals, and their intracellular concentrations are in the mM range [1]. The most common polyamines are putrescine, spermidine, and spermine.

In recent years, it has become clear that polyamines contribute significantly to extending the healthy life span of various organisms. The first report on the extension of animal lifespan through polyamine ingestion was a 2009 study using mice [2]. The study reported that feeding diets containing high concentrations of putrescine, spermine, and spermidine increased blood polyamine levels and reduced aging in the kidneys and liver, resulting in an extension of the lifespan [2]. In the same year, experiments with *Caenorhabditis elegans*, *Drosophila melanogaster*, and mice showed that the administration of spermidine was effective in extending the lifespan, and an enhancement in histone acetylation and induction of autophagy was observed in these animals [3]. In 2011, it was reported that the administration of *Bifidobacterium* to mice increased spermidine levels in the intestinal tract and decreased haptoglobin levels, an inflammatory signal, in the urine, resulting in an extension of the lifespan [4]. Furthermore, in a 2013 study, the oral administration of polyamines suppressed inflammation and abnormal DNA methylation throughout the body in mice [5]. In mice, CD11a cells present in the blood were compared between those in the low- and high-polyamine-diet groups, revealing a significant decrease in the high-polyamine-diet group [5]. In addition, research reported in 2014 that the simultaneous oral intake of probiotics and arginine increased putrescine concentrations in the intestinal tract, which simultaneously improved brain function and reduced inflammation in the colon [6]. Furthermore, the levels of the inflammatory cytokines IL-6 and MIP-2 in the blood were significantly reduced in the group that received probiotics and arginine simultaneously [6].

The health-promoting effects of polyamines are not limited to extending life expectancy. In 2013, it was reported that the oral administration of spermidine to 30-day-old *D. melanogaster* inhibited age-related memory loss and promoted autophagy in the brain [7]. In addition, a 2016 study showed that mice orally administered spermidine had improved cardiac function, increased lifespan, and induced autophagy in cardiomyocytes [8]. Furthermore, in a 2021 study, gnotobiotic mice were inoculated with wild-type *Escherichia coli* or a polyamine-biosynthesis-deficient isogenic mutant (Δ*speAB* Δ*speC* Δ*speF*), and the results showed that polyamines derived from gut bacteria were important for the proliferation of intestinal epithelial cells and the proper differentiation of macrophages in the mouse colon [9]. Moreover, in mice with experimentally induced colitis, we compared those with established wild-type *E. coli* to those with established polyamine-non-producing bacteria. The results showed a decrease in colitis pathology scores and a significant increase in survival rates [9]. The most recent study, conducted in 2022, reported that supplying spermidine to T cells in ageing mice with mitochondrial failure restored and enhanced T cell mitochondrial function by binding to the mitochondrial trifunctional protein, which is responsible for fatty acid oxidation in T cell mitochondria [10].

Polyamines are essential for cell proliferation and are present in high concentrations in actively proliferating cells [11], such as cancer cells [12]. In recent years, attempts have been made to treat cancer by lowering elevated polyamine levels in cancer cells, and some of these attempts have advanced to clinical trials [13].

Polyamines present in the colonic lumen are derived from gut bacteria [14] and are reported to be absorbed by the host through the colonic epithelium [6,9], making it one of the most important sources of polyamines for the host. In order to optimize polyamine concentrations in the human intestinal lumen, it is necessary to elucidate the mechanism of polyamine biosynthesis in the gut microbiota. To understand this mechanism, it is important to determine the polyamine metabolic pathways and transporters, and these have been extensively studied in the model intestinal bacterium *E. coli* [15,16].

Putrescine is synthesized in *E. coli* cells through two different pathways. The first pathway involves the conversion of ornithine to putrescine by the enzyme ornithine decarboxylase (SpeC). The second pathway involves the sequential reaction of two enzymes, arginine decarboxylase (SpeA) and agmatinase (SpeB) [17], which convert arginine to putrescine. Furthermore, putrescine is converted to spermidine, another polyamine, through the action of spermidine synthase (SpeE) [18]. In this reaction, an aminopropyl group derived from decarboxylated *S*-adenosylmethionine, which is generated by the reaction catalyzed by *S*-adenosylmethionine decarboxylase (SpeD), is transferred to putrescine.

Four transporters that can take up extracellular putrescine into *E. coli* cells have been identified. PotFGHI [19] is an ATP-dependent putrescine transporter that belongs to the ATP-binding cassette (ABC) transporter family and PotABCD [20] is a spermidine transporter and is also a member of the ABC transporter family, but it can take up putrescine with lower affinity. PuuP [21] and PlaP [22] are putrescine transporters that utilize the proton-motive force. SapBCDF [23] is an ABC transporter that exports putrescine out of the bacterial cell. PotE is active in both the uptake and export of putrescine [24,25]. Then, export occurs through a putrescine–ornithine antiporter activity [24], while the uptake is dependent on the membrane potential [25]. The spermidine transporter MdtJI has also been reported in *E. coli* to prevent toxicity from the accumulation of excess spermidine in the bacteria [26].

*E. coli* has two pathways to metabolize putrescine to succinate via GABA. The first is a pathway with γ-aminobutyraldehyde as a reaction intermediate. In this pathway, putrescine is metabolized to GABA without γ-glutamylation [27]. One other pathway is called the Puu pathway [28], in which putrescine is γ-glutamylated and metabolized to γ-Glu-GABA via γ-glutamyl-γ-aminobutyraldehyde, followed by hydrolysis of the γ-glutamyl bond to GABA and Glu by PuuD [29]. In this pathway, putrescine in the medium is imported to the cell through the transporter PuuP [21]. First, PuuA uses ATP to bind glutamate to one amino group of putrescine, producing γ-glutamylputrescine [30]. Next, γ-glutamylputrescine is oxidized by PuuB to γ-glutamylγ-aminobutyraldehyde. It is then believed to be further oxidized by PuuC to γ-glutamyl-GABA. The γ-glutamyl group is then cleaved by PuuD, releasing glutamate and GABA [31]. GABA is then deaminated by PuuE to form succinate semialdehyde [32]. Finally, succinate semialdehyde is oxidized by YneI to form succinate [32].

In contrast to polyamine metabolic pathways and transporters being well studied in *E. coli*, which is not the dominant species in the human gut, few studies have examined polyamine biosynthetic pathways in the predominant gut bacterial species. Furthermore, the polyamine biosynthetic pathway in *E. coli* differs from the pathway predicted to be present in the most predominant gut microbiota species [33]. In *Enterococcus faecalis*, ranked 54th among the 56 most abundant species of commensal gut microbiota in Europeans [34], an agmatine–putrescine antiporter (AguD [35]) takes up agmatine into bacterial cells. Then, agmatine is hydrolyzed into *N*-carbamoylputrescine and ammonia-catalyzed by agmatine deiminase (AguA [36]), and *N*-carbamoylputrescine is converted to putrescine via a reaction catalyzed by putrescine transcarbamylase (AguB [36]). Putrescine produced from a series of biosynthetic pathways within the bacterial cell is exported by AguD to the outside of the bacterial cell [35].

*Bacteroides thetaiotaomicron* is ranked 8th among the 56 most abundant species of commensal gut microbiota in Europeans [34]. Phylogenetically, the phylum *Bacteroidota* accounts for approximately 43% of the 56 most abundant species of human commensal gut microbiota, and the genus *Bacteroides* accounts for approximately 36% [34]. Therefore, analysis of *B. thetaiotaomicron* can significantly help to understand polyamine metabolism throughout the human commensal gut microbiota. In addition, *B. thetaiotaomicron* has been reported to possess anti-inflammatory properties, enhance mucosal barrier function, and restrict pathogen invasion [37]. The administration of *B. thetaiotaomicron* in autoimmune inflammatory bowel disease mouse models protects against weight loss, histological changes in the colon, and inflammatory markers [38]. *B. thetaiotaomicron* has been reported to induce NF-κB-relaxed aspartate-auxotrophic-PPARγ complexes in colon cancer cell line (Caco-2) cells in vitro and to downregulate NF-κB-induced inflammatory genes such as TNFα [39]. Furthermore, the NF-κB pathway regulates T cell differentiation in asthma by controlling the expression of inflammatory genes [40], especially those encoding IL-6 and TNF-α, suggesting the potential role of *B. thetaiotaomicron* in this regulation [41]. However, the genus *Bacteroides* has been reported to have potentially detrimental effects on health. The proportion of genus *Bacteroides* increased in the gut microbiota of immigrants to the U.S., and a decrease in bacterial enzymes is related to the breakdown of plant fibre and obesity [42]. In addition, the *Bacteroides* enterotype is more common in patients with depression [43].

**Figure 1:**
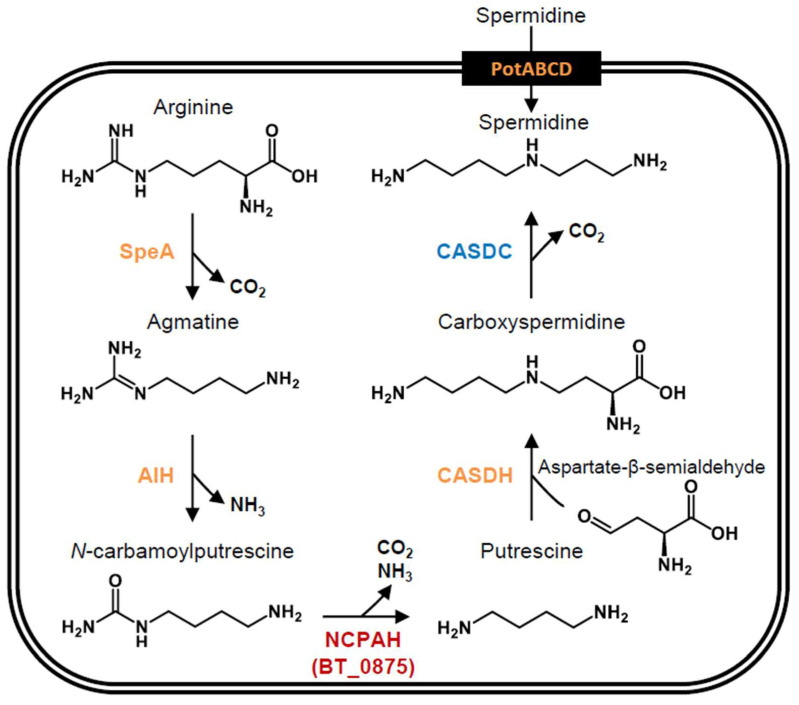
Polyamine biosynthetic pathway in *B. thetaiotaomicron*. The pathway is shown with the corresponding enzymes, which were predicted by BLASTP analyses in our previous study [45]. NCPAH, the enzyme analyzed in this study, is indicated by red characters, while the enzyme analyzed in our previous study is shown by blue characters. The other predicted enzymes are shown by orange characters. The abbreviations are as follows. SpeA: arginine decarboxylase; AIH: agmatine deiminase/iminohydrolase; NCPAH: *N*-carbamoylputrescine amidohydrolase; CASDH, carboxyspermidine dehydrogenase; CASDC: carboxyspermidine decarboxylase; PotABCD: ATP-binding cassette transporter for spermidine.

A polyamine biosynthetic pathway via *N*-carbamoylputrescine, previously reported in *C. jejuni* [33], is predicted to be present in *B. thetaiotaomicron* by the BLAST analyses. In the predicted pathway (Figure 1) arginine is decarboxylated by SpeA to form agmatine. Next, agmatine is converted to *N*-carbamoylputrescine with the liberation of ammonia by a reaction catalyzed by agmatine iminohydrolase (AIH [33]). Then, *N*-carbamoylputrescine is converted to putrescine with the liberation of ammonia and carbon dioxide by *N*-carbamoylputrescine amidohydrolase (NCPAH [33]). The synthesized putrescine is converted to carboxyspermidine by the reductive condensation of putrescine and aspartate-β-semialdehyde catalyzed by carboxyspermidine dehydrogenase (CASDH [33]). Finally, carboxyspermidine is decarboxylated by carboxyspermidine decarboxylase (CASDC) to form spermidine. Additionally, *B. thetaiotaomicron* is also predicted to take up extracellular spermidine by PotABCD, a homolog of the *E. coli* spermidine transporter [44].

In a previous study, we revealed that *B. thetaiotaomicron* accumulates spermidine as its sole polyamine and that CASDC is essential for converting carboxyspermidine to spermidine [45], but NCPAH, which is predicted to biosynthesize putrescine, the precursor of spermidine, remains unstudied. Here, we performed biochemical and genetic analyses of predicted NCPAH of *B. thetaiotaomicron*. As the genus *Bacteroides* is predominant and represents 30% of all bacteria in the human intestinal lumen, the results of this study provide a better understanding of total gut bacterial polyamine production.

## 2. Material and Methods

### 2.1. Chemicals

Agmatine and cadaverine were purchased from Tokyo Chemical Industry (Tokyo, Japan). *N*-carbamoylputrescine was synthesized by Life Chemical (Kiev, Ukraine). The chemicals 1,4-butanediammonium dichloride and L(+)-arginine hydrochloride were purchased from Fujifilm Wako Pure Chemical (Osaka, Japan). Spermidine trihydrochloride and spermine tetrahydrochloride were purchased from Nacalai Tesque (Kyoto, Japan). All other reagents were of analytical grade.

### 2.2. Bacterial Strains, Culture, Medium

Bacterial strains, plasmids, and primers used in this study are listed in Table 1 and Table 2. *B. thetaiotaomicron* Δ*tdk* was a kind gift from Dr. Thomas J. Smith (Donald Danforth Plant Science Center, USA) and Dr. Nicole M. Koropatkin (University of Michigan Medical School, USA), while *B. thetaiotaomicron* JCM 5827^T^ was from Japan Collection of Microorganisms (Tsukuba, Japan). These strains were anoxically cultured at 37 °C in Gifu anaerobic medium (GAM; Nissui Pharmaceutical, Tokyo, Japan) or polyamine-free minimal medium (pH7.2) [45,46], in which dissolved oxygen was eliminated from the media beforehand, as in previous reports [47]. The polyamine-free minimal medium was composed of the following nutrients: 0.5% (*w*/*v*) glucose, 100 mM KH_2_PO_4_, 15 mM NaCl, 8.5 mM (NH_4_)_2_SO_4_, 4 mM l-cysteine, 1.9 μM hematin, 200 μM l-histidine, 1 μg/mL vitamin K_3_, 5 ng/mL vitamin B_12_, 100 μM MgCl_2_, 1.4 μM FeSO_4_, and 50 μM CaCl_2_ [45]. The anoxic culture was conducted in an InvivO_2_ 400 chamber (10% H_2_, 10% CO_2_, and 80% N_2_; Ruskinn Technology, Bridgend, UK). *E. coli* strains DH5α and CC118 λ*pir* were used for genetic manipulation, while S17-1 λ*pir* was used as a donor host in bacterial conjugation. *E. coli* was cultured at 37 °C in Luria–Bertani (LB) medium. Where necessary, ampicillin (final concentration: 100 μg/mL), chloramphenicol (15 μg/mL), erythromycin (25 μg/mL), gentamycin (200 μg/mL), and 5-fluoro-2′-deoxyuridine (200 μg/mL) were added to the media.

### 2.3. Disruption and Complementation of Ncpah in B. thetaiotaomicron

Gene disruption of *ncpah* in *B. thetaiotaomicron* was performed using the previously established method [46]. DNA cloning was conducted with the In-Fusion cloning HD kit (Takara Bio USA, Mountain View, San Jose, CA, USA). The upstream and downstream regions (750 bp each) of *ncpah* were PCR-amplified from the JCM 5827^T^ genome as template using the primer pairs Pr-MS46/47 and Pr-MS48/49, respectively. The resulting two DNA fragments were ligated by overlap PCR using a primer pair Pr-MS46/49 and inserted into the SalI site of pExchange-*tdk* [46]. The resulting plasmid pMSK5 of MS108 was transferred by bacterial conjugation to *B. thetaiotaomicron* Δ*tdk*, and then *ncpah* knockout (Δ*ncpah*) was obtained by the double-crossover event as described previously [45,46]. Introduction of the gene disruption into the target locus was verified by Sanger sequencing of DNA fragment PCR-amplified from the genome of the disruptant as template.

The *ncpah*-complemented strain was generated as follows. The *rpoD* (sigma 70 factor gene) promoter and *ncpah* gene were PCR-amplified from JCM 5827^T^ genome as template using the primer pairs Pr-MS52/53 and Pr-MS50/51, respectively. These two DNA fragments were inserted into the PstI and NotI site of pNBU2-*bla*-*ermG*b [46], and then the resulting plasmid pMSK6 of MS110 was inserted into the NBU2 *att1* site on the chromosome of *B. thetaiotaomicron* Δ*tdk* Δ*ncpah* as described previously [45,46] to obtain the *ncpah*-complemented strain (Δ*ncpah att1*::*ncpah^+^*). The insertion of the plasmid into the targeted locus was verified by genomic PCR.

### 2.4. High-Performance Liquid Chromatography (HPLC) Analysis of Polyamines in Cells and Culture Supernatant

Polyamines in the cells and culture supernatant of *B. thetaiotaomicron* were analyzed as reported previously [45]. Specifically, cells of *B. thetaiotaomicron* strains (parental strain, Δ*ncpah*, and Δ*ncpah att1*::*ncpah^+^*) were grown overnight in liquid GAM and harvested with centrifugation at 3400× *g* for 3 min. After washing once with the polyamine-free minimal medium, the cells were inoculated into 20 mL of the same fresh minimal medium to give an initial optical density at 600 nm (OD_600_) of 0.03. The bacterial strains were then grown at 37 °C for 30 h, during which the growth was monitored by measuring OD_600_ with a spectrophotometer. Cultures were collected at the appropriate times, after which cells and supernatants were obtained by centrifugation at 18,700× *g* for 5 min at 4 °C.

The cells and supernatants were used for polyamine analysis. Supernatants were mixed with trichloroacetic acid at a final concentration of 10% (*w*/*v*) and centrifuged twice at 18,700× *g* for 5 min at 4 °C, after which the resulting supernatants were filtered through a Cosmonice filter W (Nacalai Tesque Inc., Kyoto, Japan) and used for subsequent HPLC analysis. Similarly, the cells were washed once with phosphate-buffered saline (18,700× *g*, 4 °C, 5 min), resuspended in 300 μL of 5% (*w*/*v*) trichloroacetic acid, and incubated in boiling water for 15 min. The samples were then centrifuged at 18,700× *g*, 4 °C for 5 min to separate cell debris and supernatants, the latter of which were filtered through a Cosmonice filter W (Nacalai Tesque Inc., Kyoto, Japan) and used for subsequent HPLC analysis. Cell debris, which was dissolved in 300 μL of 0.1 N NaOH, was used to measure protein concentration by the Bradford method using bovine serum albumin as a standard (Bio-Rad protein assay kit; Bio-Rad Laboratories, Inc., Hercules, CA, USA).

For HPLC analysis, a cation exchange column (#2619PH, 4.6 × 50 mm; Hitachi, Tokyo, Japan) was used as in our previous report [45]. The polyamines were derivatized with *o*-phthalaldehyde with the postcolumn method and were detected with a fluorescence detector at λ_ex_ 340 nm and λ_em_ 435 nm. The concentration of each polyamine was calculated based on a standard curve created using standards of known concentrations. The standards used and their retention times were as follows: agmatine, 33.7 min; cadaverine, 20.5 min; *N*-carbamoylputrescine, 6.2 min; putrescine, 15.2 min; spermidine, 26.0 min; and spermine, 38.1 min. As a result, the concentration of polyamines in the culture supernatant was shown as μM, while that of intracellular polyamines was expressed as nmol/mg cellular protein.

### 2.5. Expression, Purification, and Characterization of Recombinant NCPAH

Recombinant NCPAH of *B. thetaiotaomicron* was expressed as a C-terminal His_6_-tagged form. The *ncpah* gene was PCR-amplified from JCM 5827^T^ genome as template using the primer pair Pr-MS435/436 and inserted into the NdeI and XhoI site of pET23b (Novagen). The resulting plasmid pMSK106 was introduced into *E. coli* BL21(DE3). This strain was designated MS821.

MS821, an *E. coli* strain for His_6_-tagged NCPAH overexpression (Table 1), was grown in LB medium supplemented with ampicillin at 25 °C with shaking at 140 rpm. When OD_600_ reached to ~0.5, a final concentration of 0.1 mM isopropyl β-d-1-thiogalactopyranoside (IPTG) was added to the culture. After further 24 h incubation, the cells were harvested by centrifugation, resuspended in 50 mM potassium phosphate buffer (pH 8.0) containing 8 mM imidazole, and disrupted by sonication and centrifuged to obtain cell-free extract. The cell-free extract was applied to a Ni-NTA spin column (Qiagen, Hilden, Germany) equilibrated with lysis buffer (NPI-10 buffer containing 50 mM NaH_2_PO_4_, 300 mM NaCl, 10 mM imidazole, pH 8.0). The spin column was washed twice with wash buffer (NPI-20 buffer containing 50 mM NaH_2_PO_4_, 300 mM NaCl, and 20 mM imidazole, with pH 8.0) and eluted with elution buffer (NPI-500 buffer containing 50 mM NaH_2_PO_4_, 300 mM NaCl, 500 mM imidazole, pH 8.0) to yield recombinant NCPAH. The buffer of the resulting eluate was replaced with 50 mM potassium phosphate buffer (pH 8) using an Amicon Ultra-0.5 centrifugal filter unit (30 kDa cut off; Millipore, Billerica, MA, USA), in which addition of 400 µL of the same buffer followed by centrifugation (14,000× *g* for 10 min at 4 °C) was repeated five times. The purity of the protein was verified using SDS-polyacrylamide gel electrophoresis (Supplementary Figure S2). The protein concentration was determined with a Bradford assay using bovine serum albumin as a standard.

### 2.6. Enzymatic Assay Using Recombinant NCPAH

An enzymatic assay was performed at 50 °C for 40 min in a 400 µL reaction mixture containing 50 mM MES-NaOH buffer (pH 7.0), 20 ng/µL NCPAH, and 0–1.5 mM *N*-carbamoylputrescine. The reaction was started by adding different concentrations of *N*-carbamoylputrescine. Then, 100 µL aliquots were taken at 0, 20, and 40 min and the reactions were stopped by heating for 3 min at 95 °C. Activity was measured by quantifying ammonia released from *N*-carbamoylputrescine using indophenol blue method (calorimetric method) as described previously [48]. The standard curve was created by measuring the absorbance at 640 nm using known concentrations of NH_4_Cl. The kinetic parameters were determined by curve fitting the experimental data under different concentrations of *N*-carbamoylputrescine to Michaelis–Menten equation (GraphPad Prism v8.4.3).

The optimal temperature was determined by changing the reaction temperature within 20–60 °C. The enzymatic assay was performed for 60 min in a 400 µL reaction mixture containing 50 mM MES-NaOH buffer (pH 6.5), 20 ng/µL NCPAH, and 1 mM *N*-carbamoylputrescine, and the activity was measured with the indophenol blue method as mentioned above.

The optimal pH was determined by using different buffers (MES-NaOH buffer for pH 5.5–7.0; HEPES-NaOH buffer for pH 7.0–8.0; and TAPS-NaOH buffer for pH 8.0–9.0). The assay was conducted at 50 °C for 60 min in a 100 µL reaction mixture containing 50 mM buffer, 20 ng/µL NCPAH, and 1 mM *N*-carbamoylputrescine. The reaction was stopped by adding 100% (*w*/*v*) trichloroacetic acid to give a final concentration of 10% (*w*/*v*). After centrifugation at 21,487× *g* for 10 min, the supernatant was filtered using Cosmonice filter W (Nacalai Tesque Inc.) and subjected to HPLC, in which the activity was measured by quantifying the concentration of putrescine. The standard curve was created based on known concentrations of putrescine.

The effect of polyamines and their derivative compounds on the enzymatic activity was examined by adding 1 mM arginine, agmatine, putrescine, or spermidine into the reaction mixture. The assay was conducted at 50 °C for 40 min in a 100 µL reaction mixture containing 50 mM MES-NaOH buffer (pH 7.0), 5 ng/µL NCPAH, and 1 mM *N*-carbamoylputrescine. The activity was measured by quantifying putrescine using HPLC.

## 3. Results

### 3.1. Disruption of Ncpah Abolishes Accumulation of Intracellular Spermidine in B. thetaiotaomicron

**Figure 2:**
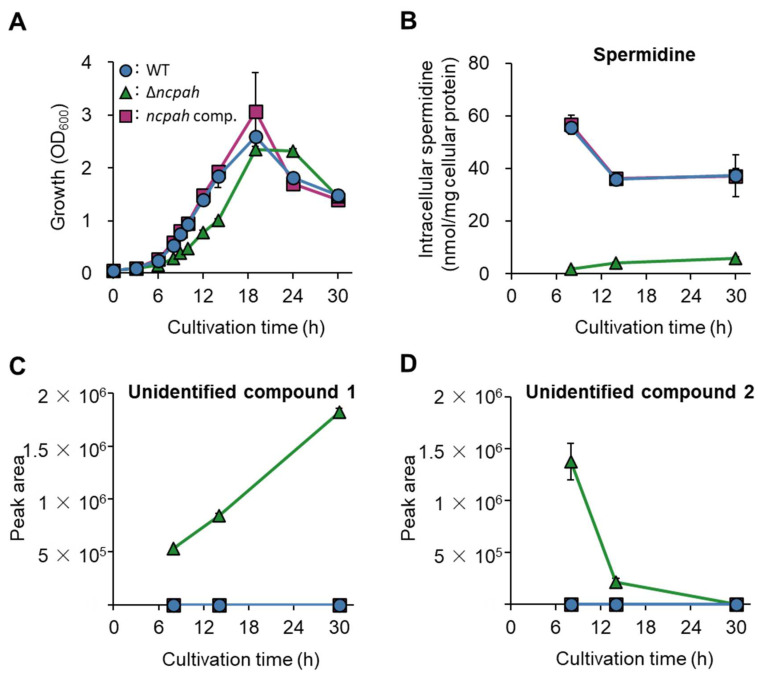
Disruption of *ncpah* affects growth and polyamine profiles in *B. thetaiotaomicron*. (**A**) Growth curve of parental strain (MS39), *ncpah* deletion strain (MS123), and *ncpah*-complemented strain (MS140) in polyamine-free minimal medium. (**B**) Intracellular spermidine concentrations at different cultivation times are shown. (**C**,**D**) The peak area of unknown amine compounds 1 and 2 present in the cells at different cultivation times are shown. The abundance of the unknown amine compounds was represented as peak areas due to lack of the appropriate standards. Circle: parental strain (MS39); triangle: Δ*ncpah* strain (MS123); square: *ncpah*-complemented strain (MS140). Data are mean ± standard deviation of biological triplicates.

To examine the physiological role of *ncpah* in the bacterial growth and polyamine production of *B. thetaiotaomicron*, we generated a *ncpah* deletion strain (Δ*ncpah*) and a *ncpah*-complemented strain of *B. thetaiotaomicron*. Growth of the Δ*ncpah* strain was slower in polyamine-free medium compared with those of parental and *ncpah*-complemented strains (Figure 2A). The generation time was longer in the Δ*ncpah* mutant strain (144.4 ± 0.3 min) compared to parental strain and *ncpah*-complemented strains (112.7 ± 0.4 and 116.8 ± 0.7 min), but was indistinguishable between the parental and *ncpah*-complemented strains. We also confirmed that the parental strain produced intracellular spermidine as the sole polyamine, and the concentration of spermidine was decreased from exponential to stationary phases (from 56.7 to 36.2 nmol/mg cellular protein) (Figure 2B; Supplementary Figure S1). While the ability of Δ*ncpah* to produce spermidine was severely decreased (<6 nmol/mg cellular protein), the complementation of *ncpah* restored the production of spermidine (36.1–55.7 nmol/mg cellular protein). These results indicate that *ncpah* is involved in spermidine biosynthesis and contributes to growth in *B. thetaiotaomicron*. Another finding was that the Δ*ncpah* strain intracellularly produced the two unidentified putative amine compounds (Figure 2C,D), which were neither *N*-carbamoylputrescine, putrescine, cadaverine, spermidine, agmatine, nor spermine (Supplementary Figure S1).

### 3.2. NCPAH Converts N-carbamoylputrescine to Putrescine and the Activity Is Regulated by Polyamines and the Polyamine Precursor Agmatine

**Figure 3:**
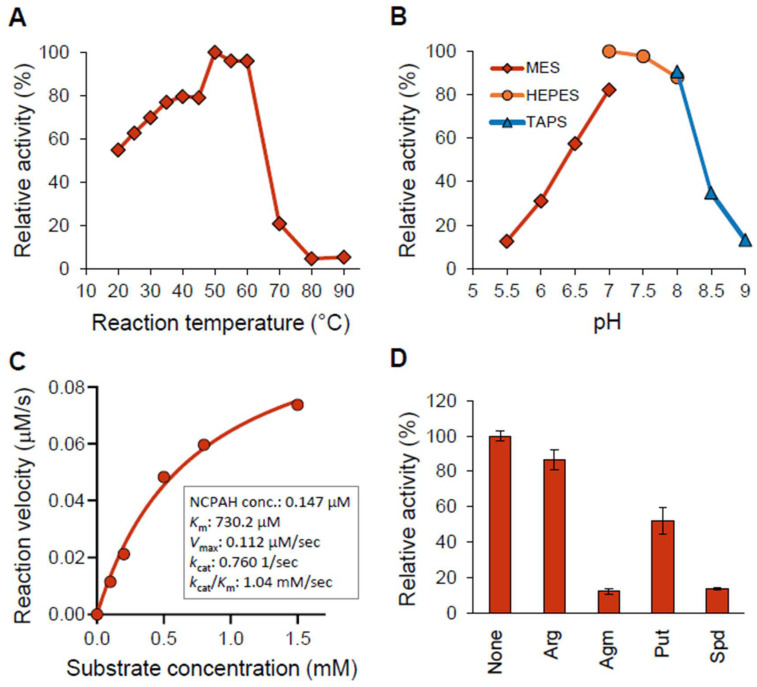
Characterization of recombinant NCPAH. (**A**) The effect of reaction temperature on activity was analyzed using the indophenol blue method. The reaction rate at 50 °C was set as 100% and the NCPAH activity at other temperatures was expressed as relative values. (**B**) The effect of pH on activity was analyzed using HPLC. The reaction rate at pH 7.0 was set as 100% and the NCPAH activity at other pH values was expressed as relative values. (**C**) Substrate–saturation curve of recombinant NCPAH with *N*-carbamoylputrescine as a substrate. The kinetic analysis was performed by varying the concentrations of *N*-carbamoylputrescine in NCPAH reactions using the indophenol blue method. (**D**) The effect of polyamines and their derivatives on activity was analyzed using HPLC. Arg: arginine; Agm: agmatine; Put: putrescine; Spd: spermidine.

NCPAH was characterized using the purified recombinant NCPAH-(His)_6_ (Supplementary Figure S2). An enzymatic assay using indophenol blue method showed that NCPAH converts *N*-carbamoylputrescine to putrescine (Supplementary Figure S3). Next, the effect of reaction temperature (20–90 °C) on the enzymatic activity was examined using indophenol blue method, and the result showed the optimal temperature was 50 °C (Figure 3A), in which the activity was 10 µmol NH_3_/min/mg, and the enzymatic activity was decreased at over 70 °C, probably due to the heat denaturation. Additionally, the effect of pH (5.5–9.0) on the activity was examined using HPLC to measure putrescine levels, and the results showed the optimal pH was 7.0, at which the activity was 11.6 µmol putrescine/min/mg (Figure 3B), and the activity was decreased to less than 40% either under pH 6.0 or over pH 8.5. The kinetic analysis, in which the NCPAH reactions were performed with varying concentrations of *N*-carbamoylputrescine and the initial velocity of NH_3_ formation was analyzed with the indophenol blue method (Supplementary Figure S3), showed that NCPAH has a substrate–saturation curve with *N*-carbamoylputrescine as a substrate (fitting to the Michaelis–Menten equation) (Figure 3C). The Michaelis constant (*K*_m_) and turnover number (*k*_cat_) were 730 µM and 0.8 s^−1^, respectively, resulting in 1.0 s^−1^ mM ^−1^ of the catalytic activity (*k*_cat_/*K*_m_). Furthermore, the effect of polyamines and their derivatives on the enzymatic activity was examined, and the addition of agmatine and spermidine at a final concentration of 1 mM inhibited the NCPAH reaction by over 80%, with the greatest extent of inhibition. In addition, the inhibitory effect of putrescine on the NCPAH reaction is approximately 50%, and there was no obvious inhibitory effect of arginine on the NCPAH reaction (Figure 3D).

## 4. Discussion

In this study, we aimed to demonstrate the importance of NCPAH, an enzyme involved in the polyamine biosynthetic pathway in *B. thetaiotaomicron*. We cultured the parental strain, the Δ*ncpah* and the *ncpah*-complemented strain of *B. thetaiotaomicron* in polyamine-free minimal medium and compared the intracellular polyamine profiles and growth. Only spermidine was present in the parental strain (Supplementary Figure S1), which is consistent with previous studies on polyamines produced by *B. thetaiotaomicron* [45,49,50]. In contrast, intracellular spermidine was significantly reduced in the Δ*ncpah* strain, whereas reduced spermidine levels in the *ncpah*-complemented strain were restored to levels similar to those in the parental strain (Figure 2B). These results suggested that *ncpah* plays an important role in spermidine biosynthesis in *B. thetaiotaomicron*.

The activity of NCPAH of *B. thetaiotaomicron* was found to be inhibited by the addition of polyamines or the polyamine precursor agmatine belonging to the polyamine biosynthetic pathway of *B. thetaiotaomicron* (Figure 3D). Putrescine, a product of NCPAH, inhibited moderately (by approximately 50%) the activity of NCPAH. It is noteworthy that the reaction catalyzed by NCPAH was inhibited significantly more strongly (over 80%) by spermidine, the product of a two-step later reaction than by putrescine, the direct product. Threonine dehydrogenase is known to be 50% feedback inhibited by its reaction products [51]. However, NCPAH was more inhibited by the reaction product compared to threonine dehydrogenase. Note that such feedback inhibition has not been reported in previous reports on NCPAH from other organisms. For example, NCPAH from *P. aeruginosa* was not inhibited by 1 mM of arginine, ornithine, putrescine, or spermidine [52]. Of note, biochemical analyses of NCPAH have been reported in *Arabidopsis thaliana* [53], *Selenomonas ruminatium* [54], *C. jejuni* [33], and *Medicago truncatula* [55], but feedback inhibition studies of NCPAH derived from these species have not been reported. Spermidine has stronger physiological activity than putrescine and is known to have adverse effects on the organism when accumulated in excessive amounts. This suggests that feedback inhibition of *B. thetaiotaomicron* in the cells prevents the excessive accumulation of spermidine. On the other hand, the kinetic parameters of NCAPH in *B. thetaiotaomicron* are comparable to those of NCPAH in *P. aeruginosa* [52] and *Selenomonas ruminatium* [54], which have been reported in previous studies (Table 3). Taken together, the results suggest that the activity of the polyamine biosynthetic enzyme NCPAH, whose enzyme activity is comparable to NCPAH in other organisms, is tightly controlled by intracellular polyamines and the polyamine precursor agmatine, supporting the existence of an in vivo polyamine homeostasis employing a feedback mechanism so far identified only in *B. thetaiotaomicron* cells.

*ncpah* deletion strains accumulated two unidentified compounds whose retention times differed from those of *N*-carbamoylputrescine, putrescine, agmatine, carboxyspermidine, spermidine, and spermine (Supplementary Figure S1). These are not found in the parental and *ncpah* complementary strains. The purification and structural determination of these molecules could lead to the identification of a novel spermidine biosynthetic pathway in *B. thetaiotaomicron*. In the *ncpah* deletion strain, a trace amount of spermidine was present in the cells despite the deletion of *ncpah* (Supplementary Figure S1). In this experiment, the strains were precultured in medium containing polyamines, washed once with main culture medium containing no polyamines, and then their suspensions were added to the main culture medium, so it is thought that almost no spermidine was brought in from the preculture. It is also unlikely that spermidine was brought in from cells of the *ncpah* deletion strain that were precultured in the GAM medium (containing polyamines). Since the intracellular spermidine concentration of *ncpah* deletion strains precultured in the GAM medium was unknown, we used the intracellular spermidine concentration of wild-type *B. thetaiotaomicron* when cultured in the GAM medium for 24 h [56] for discussion. For the washed bacterial suspension obtained from the preculture solution, the spermidine in the bacteria corresponding to OD_600_ = 0.03, the turbidity of the first outbreak of the main culture, was roughly estimated to be 0.47 nmol/mg. In contrast, the intracellular spermidine concentrations found in the *ncpah* deletion strains ranged from 2 nmol/mg to 6 nmol/mg, suggesting that more spermidine was detected in the *ncpah* deletion strains than was brought in from the preculture. It has been reported that *P. aeruginosa ncpah* mutants grew slightly on media supplemented with *N*-carbamoylputrescine as the sole carbon source [57]. Furthermore, another paper reported that crude extracts of *ncpah* mutants exhibit a slight *N*-carbamoylputrescine amidohydrolase activity [58]. Taken together, it was suggested that an alternative pathway in *P. aeruginosa* converts *N*-carbamoylputrescine to putrescine. In fact, it has been reported that in *ncpah*-deficient strains of *P. aeruginosa*, the accumulated *N*-carbamoylputrescine induces acetylpolyamine amidohydrolase, which in turn activates a pathway that converts *N*-carbamoylputrescine, not the original substrate, into putrescine [59]. However, a homology search for acetylpolyamine amidohydrolase revealed that there is no corresponding enzyme in *B. thetaiotaomicron* (data not shown). Therefore, in *B. thetaiotaomicron*, it is suggested that there is a different pathway to that reported in *P. aeruginosa* [59], which uses *N*-carbamoylputrescine as a substrate to produce putrescine through a side reaction of acetylpolyamine amidohydrolase. It has also been reported that in *C. jejuni*, a deficiency of the enzyme CASDH, which converts putrescine to carboxyspermidine, results in a significant accumulation of the downstream product spermidine [33]. In *C. jejuni*, the deletion of *casdh* is thought to activate the alternative pathway, and similarly, in *B. thetaiotaomicron*, the subject of this paper, the deletion of *ncpah* may activate the alternative pathway. This activation could be due to unidentified compound(s) that accumulate in *ncpah*-deficient strains.

*B. thetaiotaomicron* does not export polyamines in vitro [45]; there are no data on the production of polyamines in the gut. To clarify this, it is necessary to generate gnotobiotic mice monocolonized with *B. thetaiotaomicron* and analyse the production of polyamines by *B. thetaiotaomicron* in the intestinal lumen.

## 5. Conclusions

Δ*ncpah* and complemented strains were generated, and the intracellular polyamines of these strains were cultured in a polyamine-free minimal medium. Spermidine, which was detected in the parental and complemented strains, was depleted in the gene deletion strain. The purified NCPAH-(His)_6_ was characterised and observed to be capable of converting *N*-carbamoylputrescine to putrescine. In addition, we observed that agmatine and spermidine, which are produced in the polyamine biosynthetic pathway, strongly inhibited the activity of NCPAH. This feedback inhibition is suggested to regulate the reaction catalysed by NCPAH and may play a role in the intracellular polyamine homeostasis of *B. thetaiotaomicron*. Bacteria of the genus *Bacteroides* occupy >30% of the lumen of the human colon. Therefore, the findings on *B. thetaiotaomicron* may explain a significant part of the polyamine dynamics in the human intestinal lumen.

## Figures and Tables

**Table 1:** Bacterial strains and plasmids used in this study.

Strain	Description	Reference or Source
*Escherichia coli*		
BL21 (DE3)	Host for protein expression	Novagen
S17-1 λ*pir*	Donor host in bacterial conjugation (for RP4 oriT/oriR6K derivative plasmids)	National BioResource Project (NIG, Japan)
MS108	pMSK5/ S17-1 λ*pir*	This study
MS110	pMSK6/ S17-1 λ*pir*	This study
MS821	pMSK106/BL21(DE3)	This study
*Bacteroides thetaiotaomicron*		
JCM5827^T^	Same strain as ATCC 29148^T^	Japan Collection of Microorganisms
MS39	ATCC 29148^T^ except Δ*tdk*, Gm^R^	[46]
MS123	MS39 except Δ*ncpah*, Gm^R^	This study
MS140	MS123 except *att1*::*ncpah*^+^, Gm^R^, Em^R^	This study
**Plasmid**		
pET23b	Plasmid for protein expression, ColE1 replicon, Ap^R^	Novagen
pExchange-*tdk*	Plasmid for gene disruption, RP4 oriT/oriR6K, *tdk*^+^, Ap^R^, Em^R^	[46]
pMSK5	Plasmid for *ncpah* disruption, derivative of pExchange-*tdk*	This study
pMSK6	Plasmid for *ncpah* complementation, derivative of p*NBU2*-*bla*-*ermG*b	This study
pMSK106	C-terminal His_6_-tagged NCPAH expression plasmid, derivative of pET23b	This study
p*NBU2*-*bla*-*ermG*b	Plasmid for gene complementation, RP4 oriT/oriR6K, Ap^R^, Em^R^	[46]

**Table 2:** Primers used in this study.

Primer	Nucleotide Sequence (5′-3′)
Pr-MS46	TAACATTCGAGTCGAggtgtgatttattgaatacgcctg
Pr-MS47	AAATAATTATTCATCcgagcagaatcacaattaatcac
Pr-MS48	gatgaataattatttaatatgctactgaaatg
Pr-MS49	TATCGATACCGTCGAttcacattcaacggctgg
Pr-MS50	ATCTGTTTTTAAAGAatgaaaaagataaaagtaggattaatc
Pr-MS51	ACCGCGGTGGCGGCCgtttatttacggagctgccaac
Pr-MS52	TGATATCGAATTCCTtgatctggaagaagcaatg
Pr-MS53	tctttaaaaacagatttggagtg
Pr-MS435	AAGGAGATATACATAtgaaaaagataaaagtagga
Pr-MS436	GGTGGTGGTGCTCGAgatccaaaaaacgtttggtg

Uppercase letters indicate the sequence for In-Fusion cloning.

**Table 3:** Summary of biochemical functions of NCPAH from *B. thetaiotaomicron* and other bacteria.

Species	Optimal Temperature (°C)	Optimal pH	*K*_m_ (mM)	*k*_cat_ (s^−1^)	*k*_cat_/*K*_m_ (mM^−1^ s^−1^)	References
*Bacteroides thetaiotaomicron*	50	7.0	0.73	0.76	1.0	This study
*Pseudomonas aeruginosa*	40	8.0	0.50	3.3	6.6	[52]
*Selenomonas ruminatium*	45	7.0	0.22	0.18	0.81	[54]

## Data Availability

The data presented in this study are available in the main article and Supplementary Materials.

## Supplementary Materials

The following supporting information can be downloaded at: https://www.mdpi.com/article/10.3390/biomedicines11041123/s1, Figure S1: Polyamine profiles in the cells of parental and Δ*ncpah* mutant strains of *B. thetaiotaomicron*; Figure S2. SDS-PAGE analysis of purified recombinant NCPAH-(His)_6_; Figure S3. Determination of reaction rates of NCPAH at different substrate concentrations.

## References

[1] Igarashi K., Kashiwagi K. (2000). Polyamines: Mysterious modulators of cellular functions. Biochem. Biophys. Res. Commun..

[2] Soda K., Dobashi Y., Kano Y., Tsujinaka S., Konishi F. (2009). Polyamine-rich food decreases age-associated pathology and mortality in aged mice. Exp. Gerontol..

[3] Eisenberg T., Knauer H., Schauer A., Buttner S., Ruckenstuhl C., Carmona-Gutierrez D., Ring J., Schroeder S., Magnes C., Antonacci L. (2009). Induction of autophagy by spermidine promotes longevity. Nat. Cell. Biol..

[4] Matsumoto M., Kurihara S., Kibe R., Ashida H., Benno Y. (2011). Longevity in mice is promoted by probiotic-induced suppression of colonic senescence dependent on upregulation of gut bacterial polyamine production. PLoS ONE.

[5] Soda K., Kano Y., Chiba F., Koizumi K., Miyaki Y. (2013). Increased polyamine intake inhibits age-associated alteration in global DNA methylation and 1,2-dimethylhydrazine-induced tumorigenesis. PLoS ONE.

[6] Kibe R., Kurihara S., Sakai Y., Suzuki H., Ooga T., Sawaki E., Muramatsu K., Nakamura A., Yamashita A., Kitada Y. (2014). Upregulation of colonic luminal polyamines produced by intestinal microbiota delays senescence in mice. Sci. Rep..

[7] Gupta V.K., Scheunemann L., Eisenberg T., Mertel S., Bhukel A., Koemans T.S., Kramer J.M., Liu K.S., Schroeder S., Stunnenberg H.G. (2013). Restoring polyamines protects from age-induced memory impairment in an autophagy-dependent manner. Nat. Neurosci..

[8] Eisenberg T., Abdellatif M., Schroeder S., Primessnig U., Stekovic S., Pendl T., Harger A., Schipke J., Zimmermann A., Schmidt A. (2016). Cardioprotection and lifespan extension by the natural polyamine spermidine. Nat. Med..

[9] Nakamura A., Kurihara S., Takahashi D., Ohashi W., Nakamura Y., Kimura S., Onuki M., Kume A., Sasazawa Y., Furusawa Y. (2021). Symbiotic polyamine metabolism regulates epithelial proliferation and macrophage differentiation in the colon. Nat. Commun..

[10] Al-Habsi M., Chamoto K., Matsumoto K., Nomura N., Zhang B., Sugiura Y., Sonomura K., Maharani A., Nakajima Y., Wu Y. (2022). Spermidine activates mitochondrial trifunctional protein and improves antitumor immunity in mice. Science.

[11] Wirth M., Schwarz C., Benson G., Horn N., Buchert R., Lange C., Kobe T., Hetzer S., Maglione M., Michael E. (2019). Effects of spermidine supplementation on cognition and biomarkers in older adults with subjective cognitive decline (SmartAge)-study protocol for a randomized controlled trial. Alzheimers Res. Ther..

[12] Gerner E.W., Meyskens F.L. (2004). Polyamines and cancer: Old molecules, new understanding. Nat. Rev. Cancer.

[13] Casero R.A., Murray Stewart T., Pegg A.E. (2018). Polyamine metabolism and cancer: Treatments, challenges and opportunities. Nat. Rev. Cancer.

[14] Matsumoto M., Kibe R., Ooga T., Aiba Y., Kurihara S., Sawaki E., Koga Y., Benno Y. (2012). Impact of intestinal microbiota on intestinal luminal metabolome. Sci. Rep..

[15] Tabor C.W., Tabor H. (1985). Polyamines in microorganisms. Microbiol. Rev..

[16] Michael A.J., Tomonobu Kusano H.S. (2015). Biosynthesis of Polyamines in Eukaryotes, Archaea, and Bacteria. Polyamines: A Universal Molecular Nexus for Growth, Survival, and Specialized Metabolism.

[17] Boyle S.M., Markham G.D., Hafner E.W., Wright J.M., Tabor H., Tabor C.W. (1984). Expression of the cloned genes encoding the putrescine biosynthetic enzymes and methionine adenosyltransferase of *Escherichia coli* (speA, speB, speC and metK). Gene.

[18] Tabor C.W., Tabor H., Xie Q.W. (1986). Spermidine synthase of Escherichia coli: Localization of the speE gene. Proc. Natl. Acad. Sci. USA.

[19] Pistocchi R., Kashiwagi K., Miyamoto S., Nukui E., Sadakata Y., Kobayashi H., Igarashi K. (1993). Characteristics of the operon for a putrescine transport system that maps at 19 minutes on the *Escherichia coli* chromosome. J. Biol. Chem..

[20] Datsenko K.A., Wanner B.L. (2000). One-step inactivation of chromosomal genes in *Escherichia coli* K-12 using PCR products. Proc. Natl. Acad. Sci. USA.

[21] Kurihara S., Tsuboi Y., Oda S., Kim H.G., Kumagai H., Suzuki H. (2009). The putrescine Importer PuuP of *Escherichia coli* K-12. J. Bacteriol..

[22] Kurihara S., Suzuki H., Oshida M., Benno Y. (2011). A novel putrescine importer required for type 1 pili-driven surface motility induced by extracellular putrescine in *Escherichia coli* K-12. J. Biol. Chem..

[23] Sugiyama Y., Nakamura A., Matsumoto M., Kanbe A., Sakanaka M., Higashi K., Igarashi K., Katayama T., Suzuki H., Kurihara S. (2016). A Novel Putrescine Exporter SapBCDF of *Escherichia coli*. J. Biol. Chem..

[24] Kashiwagi K., Miyamoto S., Suzuki F., Kobayashi H., Igarashi K. (1992). Excretion of putrescine by the putrescine-ornithine antiporter encoded by the potE gene of *Escherichia coli*. Proc. Natl. Acad. Sci. USA.

[25] Kashiwagi K., Shibuya S., Tomitori H., Kuraishi A., Igarashi K. (1997). Excretion and uptake of putrescine by the PotE protein in *Escherichia coli*. J. Biol. Chem..

[26] Higashi K., Ishigure H., Demizu R., Uemura T., Nishino K., Yamaguchi A., Kashiwagi K., Igarashi K. (2008). Identification of a spermidine excretion protein complex (MdtJI) in *Escherichia coli*. J. Bacteriol..

[27] Shaibe E., Metzer E., Halpern Y.S. (1985). Metabolic pathway for the utilization of L-arginine, L-ornithine, agmatine, and putrescine as nitrogen sources in *Escherichia coli* K-12. J. Bacteriol..

[28] Kurihara S., Oda S., Kato K., Kim H.G., Koyanagi T., Kumagai H., Suzuki H. (2005). A novel putrescine utilization pathway involves gamma-glutamylated intermediates of *Escherichia coli* K-12. J. Biol. Chem..

[29] Schneider B.L., Reitzer L. (2012). Pathway and enzyme redundancy in putrescine catabolism in *Escherichia coli*. J. Bacteriol..

[30] Kurihara S., Oda S., Tsuboi Y., Kim H.G., Oshida M., Kumagai H., Suzuki H. (2008). gamma-Glutamylputrescine synthetase in the putrescine utilization pathway of *Escherichia coli* K-12. J. Biol. Chem..

[31] Kurihara S., Oda S., Kumagai H., Suzuki H. (2006). Gamma-glutamyl-gamma-aminobutyrate hydrolase in the putrescine utilization pathway of *Escherichia coli* K-12. FEMS Microbiol. Lett..

[32] Kurihara S., Kato K., Asada K., Kumagai H., Suzuki H. (2010). A putrescine-inducible pathway comprising PuuE-YneI in which gamma-aminobutyrate is degraded into succinate in *Escherichia coli* K-12. J. Bacteriol..

[33] Hanfrey C.C., Pearson B.M., Hazeldine S., Lee J., Gaskin D.J., Woster P.M., Phillips M.A., Michael A.J. (2011). Alternative spermidine biosynthetic route is critical for growth of Campylobacter jejuni and is the dominant polyamine pathway in human gut microbiota. J. Biol. Chem..

[34] Qin J., Li R., Raes J., Arumugam M., Burgdorf K.S., Manichanh C., Nielsen T., Pons N., Levenez F., Yamada T. (2010). A human gut microbial gene catalogue established by metagenomic sequencing. Nature.

[35] Llacer J.L., Polo L.M., Tavarez S., Alarcon B., Hilario R., Rubio V. (2007). The gene cluster for agmatine catabolism of *Enterococcus faecalis*: Study of recombinant putrescine transcarbamylase and agmatine deiminase and a snapshot of agmatine deiminase catalyzing its reaction. J. Bacteriol..

[36] Suarez C., Espariz M., Blancato V.S., Magni C. (2013). Expression of the agmatine deiminase pathway in *Enterococcus faecalis* is activated by the AguR regulator and repressed by CcpA and PTS(Man) systems. PLoS ONE.

[37] Wrzosek L., Miquel S., Noordine M.L., Bouet S., Joncquel Chevalier-Curt M., Robert V., Philippe C., Bridonneau C., Cherbuy C., Robbe-Masselot C. (2013). Bacteroides thetaiotaomicron and *Faecalibacterium prausnitzii* influence the production of mucus glycans and the development of goblet cells in the colonic epithelium of a gnotobiotic model rodent. BMC Biol..

[38] Delday M., Mulder I., Logan E.T., Grant G. (2019). Bacteroides thetaiotaomicron Ameliorates Colon Inflammation in Preclinical Models of Crohn’s Disease. Inflamm. Bowel Dis..

[39] Kelly D., Campbell J.I., King T.P., Grant G., Jansson E.A., Coutts A.G., Pettersson S., Conway S. (2004). Commensal anaerobic gut bacteria attenuate inflammation by regulating nuclear-cytoplasmic shuttling of PPAR-gamma and RelA. Nat. Immunol..

[40] Mishra V., Banga J., Silveyra P. (2018). Oxidative stress and cellular pathways of asthma and inflammation: Therapeutic strategies and pharmacological targets. Pharmacol. Ther..

[41] Alsharairi N.A. (2020). The Role of Short-Chain Fatty Acids in the Interplay between a Very Low-Calorie Ketogenic Diet and the Infant Gut Microbiota and Its Therapeutic Implications for Reducing Asthma. Int. J. Mol. Sci..

[42] Vangay P., Johnson A.J., Ward T.L., Al-Ghalith G.A., Shields-Cutler R.R., Hillmann B.M., Lucas S.K., Beura L.K., Thompson E.A., Till L.M. (2018). US Immigration Westernizes the Human Gut Microbiome. Cell.

[43] Valles-Colomer M., Falony G., Darzi Y., Tigchelaar E.F., Wang J., Tito R.Y., Schiweck C., Kurilshikov A., Joossens M., Wijmenga C. (2019). The neuroactive potential of the human gut microbiota in quality of life and depression. Nat. Microbiol..

[44] Igarashi K., Kashiwagi K. (2010). Characteristics of cellular polyamine transport in prokaryotes and eukaryotes. Plant. Physiol. Biochem..

[45] Sakanaka M., Sugiyama Y., Kitakata A., Katayama T., Kurihara S. (2016). Carboxyspermidine decarboxylase of the prominent intestinal microbiota species Bacteroides thetaiotaomicron is required for spermidine biosynthesis and contributes to normal growth. Amino Acids.

[46] Koropatkin N.M., Martens E.C., Gordon J.I., Smith T.J. (2008). Starch catabolism by a prominent human gut symbiont is directed by the recognition of amylose helices. Structure.

[47] Gotoh A., Nara M., Sugiyama Y., Sakanaka M., Yachi H., Kitakata A., Nakagawa A., Minami H., Okuda S., Katoh T. (2017). Use of Gifu Anaerobic Medium for culturing 32 dominant species of human gut microbes and its evaluation based on short-chain fatty acids fermentation profiles. Biosci. Biotechnol. Biochem..

[48] Baumann S., Sander A., Gurnon J.R., Yanai-Balser G.M., Van Etten J.L., Piotrowski M. (2007). Chlorella viruses contain genes encoding a complete polyamine biosynthetic pathway. Virology.

[49] Hosoya R., Hamana K. (2004). Distribution of two triamines, spermidine and homospermidine, and an aromatic amine, 2-phenylethylamine, within the phylum Bacteroidetes. J. Gen. Appl. Microbiol..

[50] Hamana K., Itoh T., Benno Y., Hayashi H. (2008). Polyamine distribution profiles of new members of the phylum Bacteroidetes. J. Gen. Appl. Microbiol..

[51] Bell S.C., Turner J.M. (1977). Bacterial catabolism of threonine. Threonine degradation initiated by l-threonine hydrolyase (deaminating) in a species of Corynebacterium. Biochem. J..

[52] Nakada Y., Itoh Y. (2003). Identification of the putrescine biosynthetic genes in Pseudomonas aeruginosa and characterization of agmatine deiminase and *N*-carbamoylputrescine amidohydrolase of the arginine decarboxylase pathway. Microbiology.

[53] Piotrowski M., Janowitz T., Kneifel H. (2003). Plant C-N hydrolases and the identification of a plant *N*-carbamoylputrescine amidohydrolase involved in polyamine biosynthesis. J. Biol. Chem..

[54] Liao S., Poonpairoj P., Ko K.C., Takatuska Y., Yamaguchi Y., Abe N., Kaneko J., Kamio Y. (2008). Occurrence of agmatine pathway for putrescine synthesis in *Selenomonas ruminatium*. Biosci. Biotechnol. Biochem..

[55] Sekula B., Ruszkowski M., Malinska M., Dauter Z. (2016). Structural Investigations of *N*-carbamoylputrescine Amidohydrolase from *Medicago truncatula*: Insights into the Ultimate Step of Putrescine Biosynthesis in Plants. Front. Plant. Sci..

[56] Sugiyama Y., Nara M., Sakanaka M., Gotoh A., Kitakata A., Okuda S., Kurihara S. (2017). Comprehensive analysis of polyamine transport and biosynthesis in the dominant human gut bacteria: Potential presence of novel polyamine metabolism and transport genes. Int. J. Biochem. Cell. Biol..

[57] Nakada Y., Jiang Y., Nishijyo T., Itoh Y., Lu C.D. (2001). Molecular characterization and regulation of the aguBA operon, responsible for agmatine utilization in *Pseudomonas aeruginosa* PAO1. J. Bacteriol..

[58] Haas D., Matsumoto H., Moretti P., Stalon V., Mercenier A. (1984). Arginine degradation in *Pseudomonas aeruginosa* mutants blocked in two arginine catabolic pathways. Mol. Gen. Genet..

[59] Chou H.T., Kwon D.H., Hegazy M., Lu C.D. (2008). Transcriptome analysis of agmatine and putrescine catabolism in *Pseudomonas aeruginosa* PAO1. J. Bacteriol..

