# Supplementary material for: N-Carbamoylputrescine Amidohydrolase of Bacteroides thetaiotaomicron, a Dominant Species of the Human Gut Microbiota
Source: Biomedicines. 2023 Apr 7;11(4):1123. doi: 10.3390/biomedicines11041123 (PMC10135574; doi:10.3390/biomedicines11041123)
Supplement: Supplementary file 1 [file biomedicines-11-01123-s001.zip › biomedicines-2259967-supplementary.pdf]

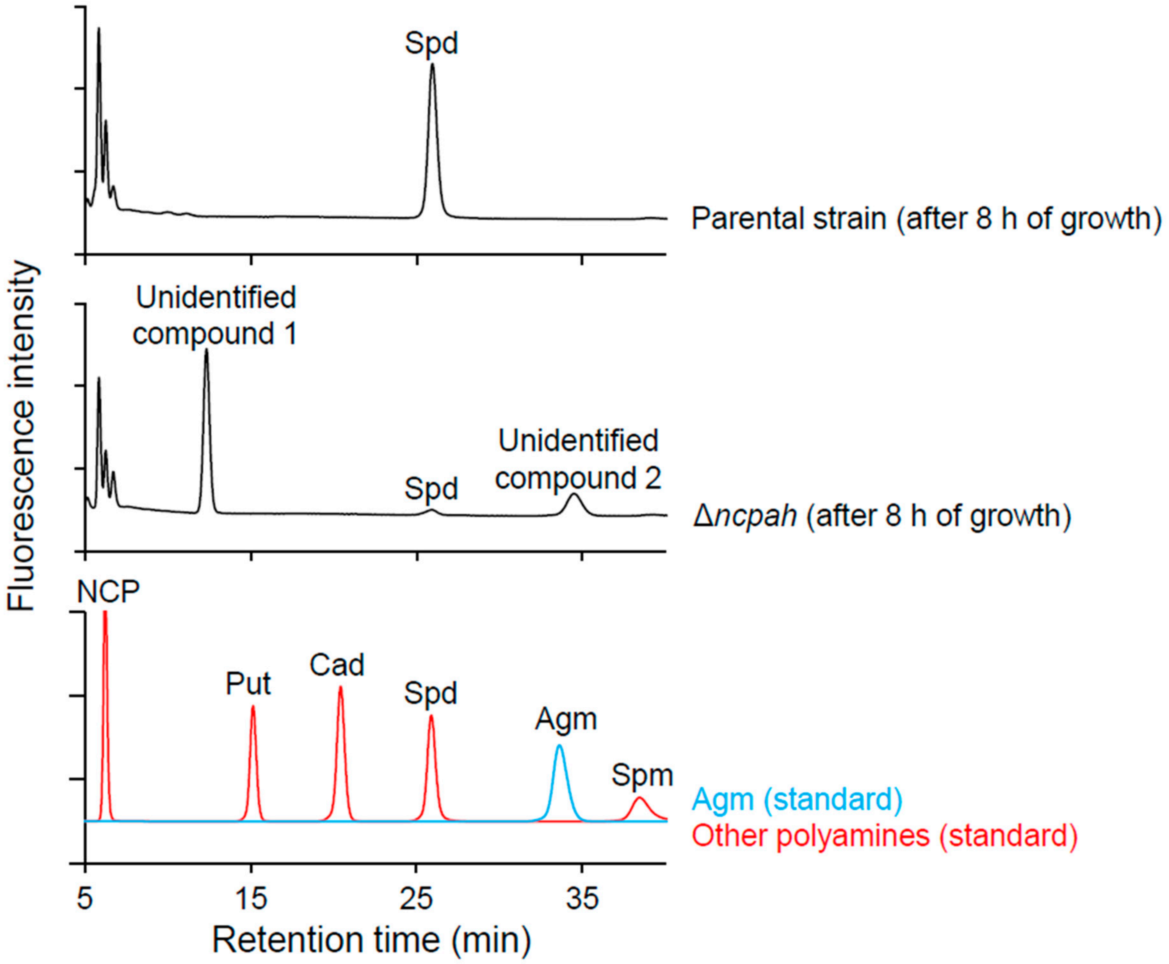

Figure S1. Polyamine profiles in the cells of parental and Δ*ncpah* mutant strains of *B. thetaiotaomicron*. HPLC chromatograms of the intracellular polyamines of the strains grown on polyamine-free minimal medium for 8 hours. Upper panel, parental strain (MS39); middle panel, Δ*ncpah* (MS123); bottom panel, polyamine standards. Agm: agmatine; NCP: *N*-carbamoylputrescine; Put: putrescine; Cad: cadaverine; Spd: spermidine; Spm: Spermine.

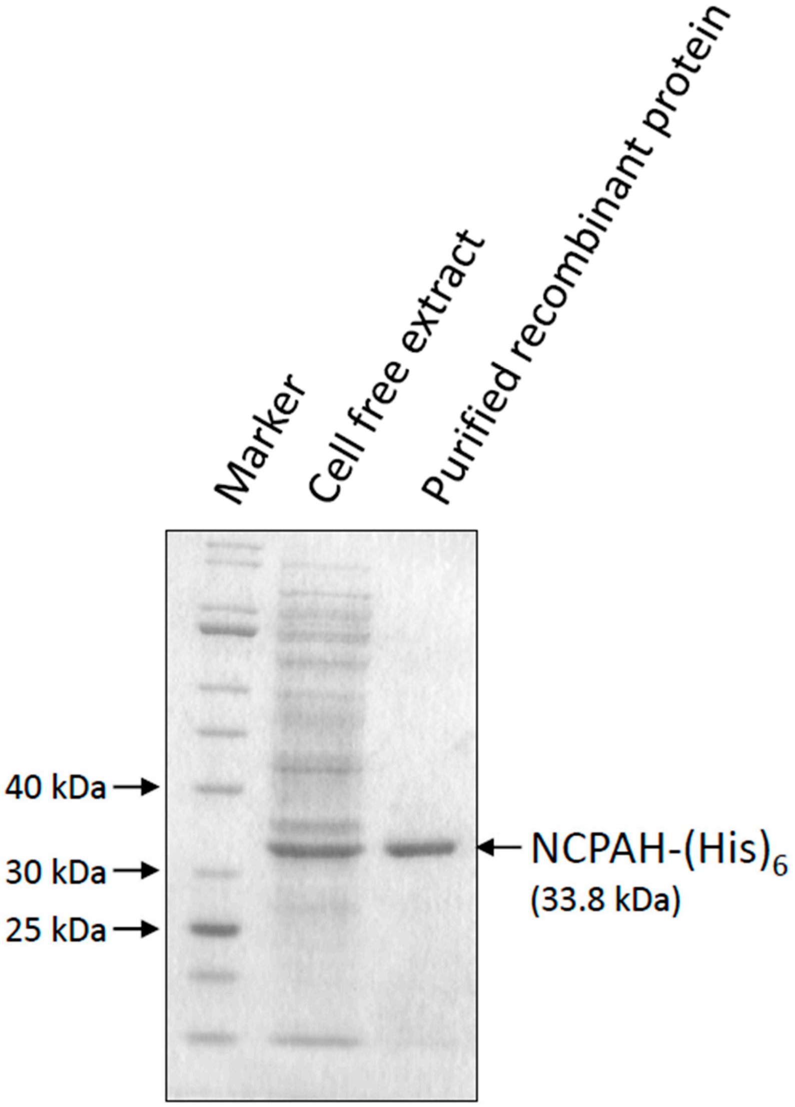

Figure S2. SDS-PAGE analysis of purified recombinant NCPAH-(His)$_6$. Cell-free extract (5 μg) and purified recombinant NCPAH-(His)$_6$ (1 μg) were loaded onto an SDS-PAGE gel, after which the gel was stained with Coomassie Brilliant Blue G-250. The estimated molecular weight of NCPAH-(His)$_6$ is 33,782.17 Da.

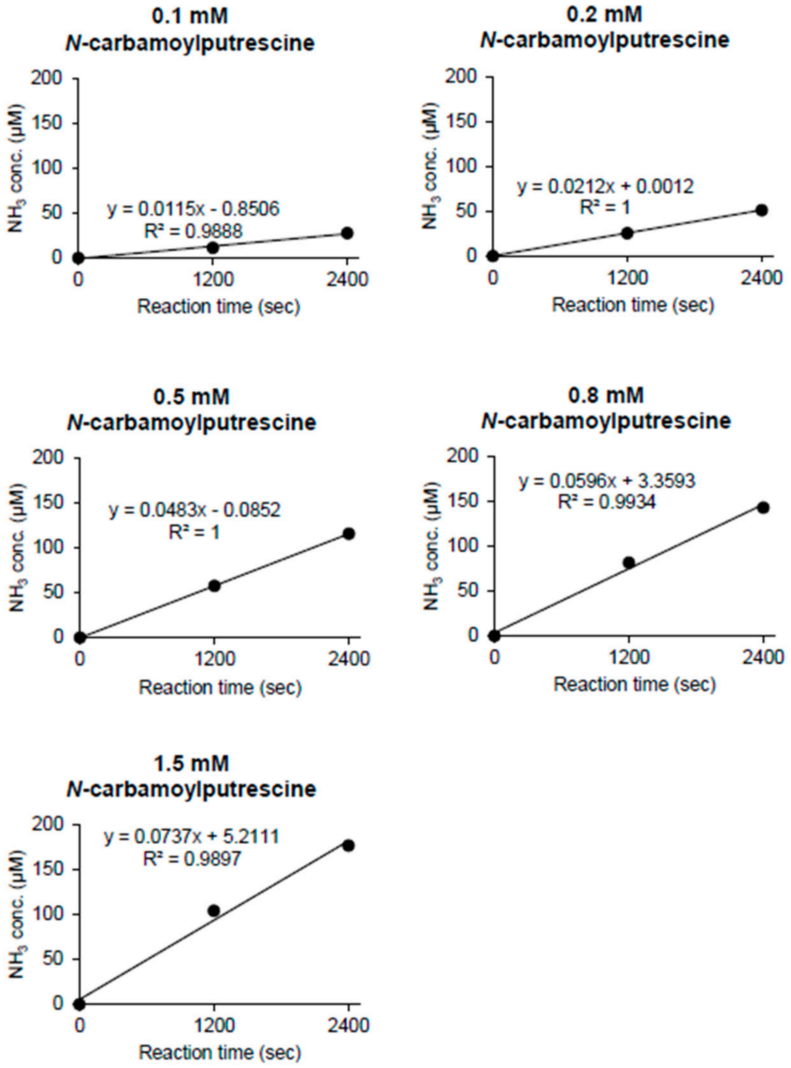

Figure S3. Determination of reaction rates of NCPAH at different substrate concentrations. The enzymatic reactions with NCPAH were conducted using different concentrations of *N*-carbamoylputrescine as a substrate, and subsequently the rate of NH_3 formation was analyzed by indophenol blue method.
